# Genomic Prediction Accounting for Residual Heteroskedasticity

**DOI:** 10.1534/g3.115.022897

**Published:** 2015-11-10

**Authors:** Zhining Ou, Robert J. Tempelman, Juan P. Steibel, Catherine W. Ernst, Ronald O. Bates, Nora M. Bello

**Affiliations:** * Department of Statistics, Kansas State University, Manhattan, Kansas 66506; † Department of Animal Science, Michigan State University, East Lansing, Michigan 48824; ‡ Department of Fisheries and Wildlife, Michigan State University, East Lansing, Michigan 48824

**Keywords:** whole-genome prediction, heteroskedastic errors, genomic breeding values, hierarchical Bayesian model, genPred, shared data resource

## Abstract

Whole-genome prediction (WGP) models that use single-nucleotide polymorphism marker information to predict genetic merit of animals and plants typically assume homogeneous residual variance. However, variability is often heterogeneous across agricultural production systems and may subsequently bias WGP-based inferences. This study extends classical WGP models based on normality, heavy-tailed specifications and variable selection to explicitly account for environmentally-driven residual heteroskedasticity under a hierarchical Bayesian mixed-models framework. WGP models assuming homogeneous or heterogeneous residual variances were fitted to training data generated under simulation scenarios reflecting a gradient of increasing heteroskedasticity. Model fit was based on pseudo-Bayes factors and also on prediction accuracy of genomic breeding values computed on a validation data subset one generation removed from the simulated training dataset. Homogeneous *vs.* heterogeneous residual variance WGP models were also fitted to two quantitative traits, namely 45-min postmortem carcass temperature and loin muscle pH, recorded in a swine resource population dataset prescreened for high and mild residual heteroskedasticity, respectively. Fit of competing WGP models was compared using pseudo-Bayes factors. Predictive ability, defined as the correlation between predicted and observed phenotypes in validation sets of a five-fold cross-validation was also computed. Heteroskedastic error WGP models showed improved model fit and enhanced prediction accuracy compared to homoskedastic error WGP models although the magnitude of the improvement was small (less than two percentage points net gain in prediction accuracy). Nevertheless, accounting for residual heteroskedasticity did improve accuracy of selection, especially on individuals of extreme genetic merit.

Use of whole-genome prediction (WGP) models to predict individual genetic merit in complex traits is being increasingly utilized in modern animal, plant and human genetics. By incorporating genotypic information from single-nucleotide polymorphism (SNP) markers, WGP models can enhance accuracies on genetic merit prediction compared to the use of pedigree information alone (Meuwissen *et al.* 2001; de los Campos *et al.* 2013a). Currently popular WGP models include ridge-regression best linear unbiased prediction (RR-BLUP), BayesA and BayesB, all proposed by Meuwissen *et al.* (2001), and subsequently modified or extended to a wide array of models collectively dubbed as Bayesian alphabet models (Habier *et al.* 2011; Gianola 2013). Typically, these Bayesian models specify either heavy-tailed distributions (*i.e.*, BayesA), variable selection (BayesCπ), or both (BayesB) on the distribution of the SNP effects.

An often underappreciated, though pervasive, assumption underlying classical WGP models across the “Bayesian alphabet” (Gianola *et al.* 2009) is that of homogeneous residual variances, often referred to as residual homoskedasticity. Yet, heterogeneity of residual variances across environments, or residual heteroskedasticity, is a well-documented phenomenon in livestock production systems (Cardoso *et al.* 2005, 2007; Kizilkaya and Tempelman 2005; Bello *et al.* 2012; Cernicchiaro *et al.* 2013), thereby raising concern about the implications of the residual homoskedasticity assumption almost universally assumed in current WGP models (Gianola and Rosa 2015). Indeed, the use of heteroskedastic models for genetic evaluations dates back to work by Foulley and Gianola (1996), who first modeled the logarithm of residual variances as a linear function of fixed effects. SanCristobal-Gaudy *et al.* (2001) presented further extensions to incorporate random effects, including correlated genetic effects, followed by a unifying framework for the structural modeling of heterogeneous variances proposed by Kizilkaya and Tempelman (2005). With the advent of genomic selection, Yang *et al.* (2011) was among the first to propose a WGP model with heterogeneous residual variances for livestock populations, though only a genetic component was specified on both mean and residual variance.

Environmentally-driven heteroskedasticity has been shown to have practical implications for the prediction of genetic merit. Hill (1984) demonstrated that proportionally more individuals were likely to be selected from more variable groups if substantial heteroskedasticity was ignored using homoskedastic error models, especially if selection pressure was intense. Early attempts to remedy this problem included the preadjustment of phenotypes, *e.g.*, by centering and scaling (Hill 1984). More modern approaches include explicit specification and modeling of sources of heteroskedasticity. For instance, Kizilkaya and Tempelman (2005) showed improved precision in estimated sire genetic merit for a birth weight trait when residual variance was specified as a function of sex and herds, finding that estimates of residual variances differed by as much as 20 times across herds.

The objectives of this study were 1) to extend classical parametric WGP models, specifically RR-BLUP, BayesA, BayesB and BayesCπ, to explicitly account for residual heteroskedasticity, and 2) to assess potential gains in prediction accuracy by explicit modeling of residual variances as a function of various environmental or management factors in simulated and actual livestock performance data.

We first introduce extensions to classical WGP models to accommodate heteroskedasticity, including a delineation of the criteria used for model performance. We then describe and present comparisons based on a simulation study and also an application to carcass traits data from pigs.

## Materials and Methods

### Classical WGP models

The classical base WGP model expresses the phenotype *y_i_* of an individual *i* (*i* *=* 1, 2,…,*n)* as a linear regression function of SNP marker effects, as follows:

$$y_{i}=x_{i}^{’}\beta +\sum_{j=1}^{p}z_{ij}g_{j}+e_{i}$$ (1)

where **β** is a vector of unknown fixed effects connected to the phenotypes via a known design vector $x_{i}^{’}$; $g_{j}$ is the unknown random effect of SNP marker *j* *=* 1, 2,…, *p* connected to $y_{i}$ via a known genotype $z_{ij}$ coded as 0, 1 or 2 to represent the dosages of the minor allele, and $e_{i}$ is the residual for animal *i*. Most current WGP models assume $e={\left\{e_{i}\right\}}_{i=1}^{n}\overset{iid}{∼}N(0,I{\text{σ}}_{e}^{2})$.

Differences between WGP models RR-BLUP, BayesA, BayesB and BayesCπ are based upon the specification of the distribution of $g={\left\{g_{j}\right\}}_{j=1}^{p}$. For RR-BLUP, $g_{j}\overset{iid}{∼}N(0,{\text{σ}}_{g}^{2})\forall j$, whereas for BayesA $g_{j}\text{|}\nu_{g},s_{g}^{2}\overset{iid}{∼}t_{\nu_{g}}\left(0,s_{g}^{2}\right)$, *i.e.*, independently and identically distributed scaled Student-*t* with common degrees of freedom $\nu_{g}$ and scale parameter $s_{g}^{2}$. This prior specification on BayesA is marginally equivalent to $g_{j}{\text{|σ}}_{g_{j}}^{2}\overset{iid}{∼}N\left(0,{\text{σ}}_{g_{j}}^{2}\right)$ such that ${\text{σ}}_{g_{j}}^{2}|\nu_{g},s_{g}^{2}∼{\text{χ}}^{\mathrm{-2}}(\nu_{g},\nu_{g}s_{g}^{2})$ with $\text{E}({\text{σ}}_{g_{j}}^{2}|\nu_{g},s_{g}^{2}\text{)}=\frac{\nu_{g}}{\nu_{g}-2}s_{g}^{2}$ (de los Campos *et al.* 2009; Yang and Tempelman 2012). BayesB further extends BayesA and specifies the distribution of $g_{j}\text{| }\nu_{g},s_{g}^{2}$ as a mixture of $t_{\nu_{g}}\left(0,s_{g}^{2}\right)$ with probability (1−π)and a point mass at 0 with probability π (Meuwissen *et al.* 2001). BayesCπ is a particular special case of BayesB with $\nu_{g}\to \infty$such that the nonzero component of the mixture is $N(0,{\text{σ}}_{g}^{2})$ (Habier *et al.* 2011).

### Heteroskedastic extension of WGP models

Following Kizilkaya and Tempelman (2005), we extend WGP models to flexibly model the residual variance ${\text{σ}}_{e}^{2}$ as a multiplicative function of both systematic and nonsystematic environmental components, thereby explicitly accounting for heteroskedasticity. Expressed in the natural logarithmic scale, this is equivalent to writing the following

$${\text{ln(σ}}_{e_{i}}^{2}\text{)}=w_{i}^{’}\text{ln(}\tau \text{)}+m_{i}^{’}\text{ln(}v\text{)}$$ , (2)

where ${\text{σ}}_{e_{i}}^{2}$ is the residual variance corresponding to the environmental or management circumstances for individual *i*; **τ** is a s×1 vector of unknown fixed effect parameters connected to ${\text{σ}}_{e_{i}}^{2}$ via a known design vector $w_{i}^{’}$ ; and similarly, **v** is a t×1 vector of unknown random effects connected to ${\text{σ}}_{e_{i}}^{2}$via a known design vector $m_{i}^{’}$. Random effects on the residual variance may include environmental effects (*i.e.*, contemporary groups), genetic effects or both. *A priori*, elements of $v={\left\{v_{l}\right\}}_{l=1}^{t}$ can be assumed independently distributed as inverted gamma $\text{IG(}\alpha_{\nu },\alpha_{\nu }-\mathrm{1)}$, $\alpha_{\nu }$>2 such that $\text{E(}v_{l}\text{)}=1$ and ${\text{σ}}_{v}^{2}=\text{Var(}v_{l}\text{)}=1/\text{(}\alpha_{v}-2\text{)}$. As a result, variation across $v_{l}$ can be characterized by defining a coefficient of variation $\text{CV}=\frac{\text{standard deviation}}{\text{mean}}=\sigma_{v}={\text{(}\alpha_{v}-2\text{)}}^{-\frac{1}{2}}$. So specified, the magnitude of the heteroskedasticity across levels of the random effect factor **v** diminishes (*i.e.*, ${\text{σ}}_{v}^{2}\to 0$) with larger values of $\alpha_{v}$(*i.e.*, $\alpha_{v}\to \infty$) (Kizilkaya and Tempelman 2005).

### Prior specifications

We specify a flat prior on **β** such that p(β)∝constant. Here,${\text{σ}}_{e}^{2}$ in the homoskedastic error model, as well as elements of **τ** in Equation (2), were specified with noninformative priors ${\text{χ}}^{-\text{2}}\mathrm{(-1,0)}$ (Gelman 2006). The hyperparameter $\alpha_{\nu }$ was assigned the vague, though proper, prior density $p(\alpha_{\nu })\propto {\left(1+\alpha_{\nu }\right)}^{-2}$, which is commonly used for strictly positive parameters (Kizilkaya and Tempelman 2005). As previously shown by Albert (1988), this prior defines a uniform prior density *U*(0,1) on the transformed variable $\varsigma =g\left(\alpha_{v}\right)={\left(1+\alpha_{v}\right)}^{-1}$. Then, by change of variables, $f_{\alpha_{v}}=f_{\varsigma }\left(g^{-1}\left(\alpha_{v}\right)\right)\left|\frac{\partial }{\partial \alpha_{v}}g^{-1}\left(\alpha_{v}\right)\right|={\left(1+\alpha_{v}\right)}^{-2},$ where *f* denotes the probability density function.

For RR-BLUP and BayesCπ, we specify ${\text{σ}}_{g}^{2}∼{\text{χ}}^{-\text{2}}\mathrm{(-1,0)}$ whereas for BayesA and BayesB, we specify ${\text{σ}}_{g_{j}}^{2}∼{\text{χ}}^{-\text{2}}(\nu_{g},\nu_{g}s_{g}^{2})$ with $\nu_{g}\mathrm{∼p}\text{(}\nu_{g}\text{)}\propto {\left(1+\nu_{g}\right)}^{\text{-}2}$ and $s_{g}^{2}∼{\text{χ}}^{-\text{2}}\mathrm{(-1,0)}$. Finally, for BayesB and BayesCπ, π is assigned a Beta(10,1) prior to reflect a relatively weak assumption that most markers have null effects for any given trait.

### Simulation study

We compared the performance of classical WGP models, namely RR-BLUP, BayesA, BayesB and BayesCπ, to that of their heteroskedastic error counterparts using a simulation study.

Ten data set replicates were each generated from base populations of 150 unrelated individuals subjected to random mating for 6000 generations. Population size was kept constant until generation 6000, after which it was expanded 10 times to 1500 individuals. The genome was composed of three chromosomes, each of length 1 Morgan, and each containing a total of 10,000 equally-spaced monomorphic loci. The number of crossover events per meiosis was simulated from a Poisson distribution with mean 1 and the location of crossover was assumed uniformly distributed in a chromosome. The mutation rate for all loci was specified to be $2.5\times 10^{-4}$per locus per generation and to be recurrent so as to ensure biallelic loci.

In Generation 6001, loci with minor allele frequency (MAF) < 0.1 or loci that failed to meet Hardy-Weinberg equilibrium based on an exact test (Wigginton *et al.* 2005) at a significant level of 0.0001 were discarded. For each dataset replicate, 60 loci were randomly selected to serve as quantitative trait loci (QTL), and an additional 3000 different loci were randomly selected to serve as SNP markers. For each of the 60 QTL, an allelic substitution effect $a_{k}$(*k* = 1, 2,…, 60) was drawn from a $t_{5}$(0,0.005), *i.e.*, a Student-*t* distribution with 0 mean and a scale of 0.005 based on five degrees of freedom. Our choice of a heavy-tailed distribution for the QTL effects is consistent with current notions of the genetic architecture of quantitative traits in livestock population, by which traits seem to be controlled by many genes of small effect and few of large effects (Hayes and Goddard 2001; Goddard *et al.* 2009). The total additive genetic variance $\sigma_{a}^{2}$ was constructed from the weighted sum of genetic variances across the QTL effects, namely ${\text{σ}}_{a}^{2}=2\sum_{k=1}^{60}q_{k}(1-q_{k})a_{k}^{2}$, where $q_{k}$ is the MAF at QTL *k*. The true breeding value (TBV) for an individual *i* was obtained as the aggregated allelic substitution effects $a_{k}$over the selected 60 QTL loci, each weighted by its corresponding allelic dosage $z_{ik}$, such that ${\text{TBV}}_{i}=\sum_{k=1}^{60}z_{ik}a_{k}$. Trait heritability was set at $h^{\text{2}}\mathrm{=0}\mathrm{.4}$.

Within each data replicate, we considered five different simulation scenarios reflecting various degrees of residual heteroskedasticity. That is, the replicated datasets described in the previous paragraph were used as blocking factors to compare scenarios across a heteroskedastic error gradient. Simulation scenarios included the case of homoskedastic residuals whereby ${\text{τ}}_{1}={\text{τ}}_{2}={\text{σ}}_{e}^{2}$ and $v_{l}=1$ for all *l* =1, 2,…, 50 levels of a random effects factor, such that ${\text{σ}}_{e_{i}}^{2}={\text{σ}}_{e}^{2}\forall i$. In this study, specification of $\alpha_{\nu }\to \infty$represents the homoskedastic error scenario, as $\sigma_{v}^{2}\to 0$. In turn, other scenarios were defined by increasing levels of residual heteroskedasticity; *i.e.*, $\alpha_{\nu }$ = 50, 12, 5, and 3, such that the standard deviations ${\text{σ}}_{v}=\sqrt{\frac{1}{\alpha_{v}-2}}$ of the relative variances ($v_{l}$) across these random effects were $\sqrt{\frac{1}{48}},\sqrt{\frac{1}{10}},\sqrt{\frac{1}{3}}\text{ and }1$, respectively. In addition, all heteroskedastic error scenarios (*i.e.*, $\alpha_{\nu }$= 50, 12, 5, and 3) further incorporated systematic sources of heterogeneity whereby ${\text{τ}}_{1}=0.8\cdot {\text{σ}}_{e}^{2*}$ and ${\text{τ}}_{2}={\text{σ}}_{e}^{2*}$, where ${\text{σ}}_{e}^{2*}$is a “fixed” reference residual variance. For data generation, the residual $e_{i}$ was sampled from $N\left(0,{\text{σ}}_{e_{i}}^{2}\right)$ where ${\text{σ}}_{e_{i}}^{2}$was obtained as a function of **τ** and **v**, as described in Equation (2). The phenotypic observation for individual *i* was generated as $y_{i}=\mu +{\text{TBV}}_{i}+e_{i}$, with μ = 3 set as a common mean for all observations. Observations from Generation 6001 were used as a training set to fit the competing WGP models and to estimate SNP effects. For each simulated dataset, individuals from Generation 6001 were randomly mated to produce Generation 6002 consisting of additional 1500 animals. Genotypes and TBV from individuals in Generation 6002 were generated to be used for validation in the simulation study. The average level of linkage disequilibrium (LD) between adjacent markers in the simulation study ranged between 0.23 to 0.25 across all replicated datasets, to represent current livestock populations (Meuwissen *et al.* 2001; Calus *et al.* 2008; Hayes *et al.* 2009; Yang *et al.* 2011).

Each replicated dataset was fitted using homoskedastic and heteroskedastic error versions of the selected WGP models, namely RR-BLUP, BayesA, BayesB and BayesCπ. Programming code needed to implement these models is available in Supporting Information, File S1. Across models, Markov Chain Monte Carlo (MCMC) was implemented with burn-in lengths of 10,000 to 35,000, followed by subsequent saving of the next 140,000 to 480,000 cycles, depending on the WGP models and diagnostics as described subsequently.

### Application to MSU swine resource population data

Data corresponding to a three-generation Duroc × Pietrain swine resource population developed at Michigan State University (MSU) was used in this study. A detailed description of the dataset is available in Edwards *et al.* (2008a, 2008b). Briefly, a total of 19 F_0_, 55 F_1_ and 928 F_2_ pigs were included in the pedigree. All F_0_ and F_1_ animals as well as 336 F_2_ animals were genotyped using the commercial Illumina PorcineSNP60 beadchip (GeneSeek a Neogen Co., Lincoln, NE) panel with a total of 62,163 SNP markers (Gualdron Duarte *et al.* 2014). Markers with more than 10% missing data, unknown physical positions, or with MAF < 0.01 were removed from further analyses. Quality control procedures followed those described in Badke *et al.* (2012). Genotypes for the remaining 592 F_2_ animals were obtained using a low-density panel of 9K tagSNP set referred to as the GeneSeek Genomic Profiler for Porcine LD (GGP-Porcine LD, GeneSeek a Neogen Company) consisting of a subset of the PorcineSNP60 panel. The F_2_ animals genotyped with the 9K low density panel were imputed to 60K with imputation accuracy of approximately 0.99, as previously described (Gualdron Duarte *et al.* 2013). From the 60K SNP, a subset of 6210 markers was selected for this study. The selected SNP subset matched the panel of 10K tagSNP previously described by Badke *et al.* (2014). Phenotypes corresponding to 29 growth traits and 25 carcass and meat quality traits were obtained for F_2_ animals, as described by Edwards *et al.* (2008a, 2008b). Traits were subjected to preliminary screening for heterogeneous residual variances using standard linear mixed models and approximately 80% of the traits showed some degree of residual heteroskedasticity. Two traits, namely carcass temperature at 45 min postmortem, and loin muscle pH at 45 min postmortem, were selected for further consideration based on potentially high and mild levels of heteroskedasticity, respectively, and were thus subjected to follow-up WGP analysis (see next section). Phenotypes for 921 and 908 F_2_ individuals were available for 45 min postmortem carcass temperature and for loin muscle pH, respectively. Phenotypes of the selected traits, genotypes and pedigree of the available animals were contained in the Supporting Information, File S2.

Each of the two selected traits were fitted using RR-BLUP, BayesA, BayesB and BayesCπ WGP models in both their homoskedastic and heteroskedastic error specifications. For both traits, **β** included the fixed effect of sex and a regression coefficient on carcass weight. The general WGP model in Equation (1) was further expanded to incorporate clustering effects of slaughter dates $d={\left\{d_{q}\right\}}_{q=1}^{33}∼N(0,{\text{σ}}_{d}^{2})$as well as polygenic effects $u\mathrm{∼N}\text{(}\mathrm{0,}A{\text{σ}}_{u}^{2}\text{)}$, where **A** is a known pedigree-based additive relationship matrix. Therefore, in our data application, the genomic expected breeding value (GEBV) for individual *i* (*i* = 1,2,…,*n*) was defined as $\sum_{j=1}^{p}z_{ij}{\hat{g}}_{j}+{\hat{u}}_{i}$. We modeled heterogeneous residual variances as presented in Equation (2), with **τ** and $v={\left\{v_{l}\right\}}_{l=1}^{33}$ specifying the fixed effects of sex and the random clustering effects of slaughter dates, respectively. Thus, the hyperparameter $\alpha_{\nu }$in $v_{l}∼\text{IG(}\alpha_{\nu },\alpha_{\nu }-1\text{)}$ reflects the magnitude of residual heteroskedasticity in the responses of interest due to slaughter dates clusters.

Prior specifications were similar to those described for the simulation study, with the following exceptions due to problems with parameter identifiability. For BayesCπ, the prior hyperparameter $\nu_{g}$ was set at $\nu_{g}$ = 3 for both traits to maximize prior uncertainty while retaining a defined mean (*i.e.*, $\nu_{g}$ > 2). Instead, the prior scale $s_{g}^{2}$ for BayesCπ was specified as $s_{g}^{\text{2}}=$ $4.95\times 10^{-7}$for carcass temperature and $s_{g}^{2}=$$2.01\times 10^{-8}$for loin muscle pH, based on the posterior medians of $s_{g}^{2}$ obtained in BayesA. For BayesB, the hyperparameter $s_{g}^{\text{2}}$ was assumed known and set at $6.35\times 10^{-7}$ for carcass temperature and $2.64\times {\text{10}}^{-8}$ for loin muscle pH, whereby these values were obtained based on the posterior median of ${\text{σ}}_{g}^{\text{2}}$ under BayesCπ with a homoskedastic error assumption. Sensitivity analyses were conducted to assess the influence of specifying $s_{g}^{2}$ on posterior inference of interest. Also due to parameter identifiability issues, the variance ${\text{σ}}_{u}^{2}$ of the polygenic effects was first estimated from traditional (*i.e.*, non-WGP) animal models that either assumed residual homo- or heteroskedasticity. These estimates of ${\text{σ}}_{u}^{2}$ under homo- and heteroskedastic assumptions were then specified as known constants when fitting homo- and heteroskedastic WGP models, respectively. Homoskedastic and heteroskedastic error specifications of the selected WGP models were fitted to each trait. In every case, a total of 20 parallel MCMC chains were run, each consisting of 12,000 to 27,000 burn-in cycles followed by 6,000 to 14,000 saved cycles. Post burn-in samples from the 20 parallel MCMC chains run on a given model can be considered samples from the joint posterior density of interest, and were thus combined for inference. Initial values of hyperparameters in each parallel chain were dispersed by an arbitrary small value while constraining them to fall within their allowable parameter space (Gelman and Rubin 1992). Posterior inference on parameters of interest was summarized for the overall dataset.

### Model comparison

For each of the WGP models considered, namely RR-BLUP, BayesA, BayesB and BayesCπ, the performance of the homoskedastic *vs.* its heteroskedastic error model counterparts was compared in both simulated data and real data using various criteria for model fit and for prediction, as follows.

First, we compared quality of global model fit using pseudo-Bayes factor (PBF) (Gelfand 1996), defined as the ratio of the conditional likelihood function under each heteroskedastic error WGP model over its homoskedastic counterpart, expressed in logarithmic scale of base 10, as follows:

$${\text{log}}_{10}{\text{ PBF}}_{\text{HT,HO}}=\sum_{i=1}^{n}{\text{log}}_{10}\text{ L(}y_{i}|y_{-i},\mathrm{HT}\text{)}-\sum_{i=1}^{n}{\text{log}}_{10}\text{ L(}y_{i}|y_{-i},\mathrm{HO}\text{)}$$ (3)

where the abbreviation HT and HO hereafter refer to the candidate heteroskedastic and homoskedastic models, respectively. Moreover, L(⋅) denotes the likelihood function of observation $y_{i}$ conditional on all remaining observations fitted with the corresponding WGP model. This conditional likelihood, also known as the conditional predictive ordinate (Gelfand 1996) for observation *i*, can be approximated by $\text{L(}y_{i}|y_{-i}\mathrm{, model}\text{)}\approx {\left(\frac{1}{B}\sum_{b=1}^{B}\frac{1}{\text{L(}y_{i}|\theta^{\left(b\right)},\mathrm{model})}\right)}^{-1}$, where *B* is the number of post burn-in MCMC iterations; $\theta^{\left(b\right)}$ represents the posterior sample for model parameters **θ** after *b* iterations post burn-in (*b =*1,2,…,*B*). A positive value of ${\text{log}}_{10}{\text{ PBF}}_{\text{HT,HO}}$ indicates support for the heteroskedastic error model based on enhanced fit to the data relative to its homoskedastic error WGP counterpart, thereby indicating evidence for heterogeneity of residual variances.

We further compared predictive performance of breeding values between competing homoskedastic and heteroskedastic error alternatives of each WGP model. For simulated data, we assess genomic prediction accuracy using the Pearson correlation between TBV in the simulated validation set and corresponding estimates ${\text{GEBV}}_{i}=\sum_{j=1}^{p}z_{ij}{\hat{g}}_{j}$, whereby ${\hat{g}}_{j}$ were obtained by fitting the WGP model to the simulated training set. Within each WGP model, we compared homoskedastic *vs.* heteroskedastic error specifications across the various scenarios using a multifactorial ANOVA on genomic prediction accuracy, with the simulated replicated dataset as a random blocking factor.

For the real data application, predictive performance was assessed using a five-fold cross-validation (Daetwyler *et al.* 2013), whereby animals within each slaughter dates cluster were randomly assigned to five nonoverlapping data partitions or folds of nearly equal size (175–191 animals). Each one of the five data folds was assigned to be a validation set exactly once. When a data fold was selected as a validation set, phenotypes of this particular fold were excluded from estimation of marker effects. Instead, phenotypes of the validation fold were predicted using estimates of SNP markers, polygenic and nongenetic effects obtained from fitting a model to the remaining data folds, referred to here at training folds. This procedure was repeated until each of the five data folds had served as a validation set once. Consequently, every phenotyped animal was excluded from estimation of marker effects once, in which case their phenotypes were predicted using estimates obtained from animals in corresponding training folds (Meuwissen *et al.* 2013). We defined cross-validation predictive ability as the Pearson correlation coefficient between observed phenotypes in the validation fold, and the corresponding predicted phenotypes from parameter estimates obtained from the training folds. That is, $\rho (y_{i},{\hat{y}}_{i})$, where $y_{i}$ and ${\hat{y}}_{i}$ are the observed and predicted phenotypes, respectively, for animal *i* in the validation fold. The predicted phenotypes ${\hat{y}}_{i}$ included estimated marker effects (weighted by their allelic frequencies) and estimated polygenic effects, as well as the estimated fixed effects of sex and carcass weight, and the random blocking effects of slaughter dates.

We also characterized potential practical implications of heteroskedasticity in the context of breeding decisions based on WGP. More specifically, we computed the Spearman’s rank correlation coefficient between GEBV from homoskedastic *vs.* heteroskedastic error specifications for the top and bottom 10% ranked individuals. Relative ranking of top and bottom 10% individuals was assessed by fitting a linear mixed model to the estimated Spearman rank correlations obtained from data replicates, and testing for differences between simulation scenarios. For real data, rank correlations of GEBV for top and bottom 10% individuals in the validation sets were compared between homoskedastic and heteroskedastic WGP models.

### MCMC diagnostics

Convergence diagnostics were implemented using the R package CODA (Plummer *et al.* 2006). We monitored convergence using trace plots. Diagnostic tests by Raftery and Lewis (1992) and by Heidelberger and Welch (1983) were conducted on the simulation study. For the data application, the Gelman and Rubin’s diagnostic on multiple MCMC chains produced a shrinkage factor < 1.2 (Colosimo and Del Castillo 2007). We also determined effective sample size (ESS) for key hyperparameters (Kass *et al.* 1998). In each case, the number of MCMC cycles was adjusted to ensure that the ESS was greater than 100 for all hyperparameters.

### Data availability

Data and R code are available in File S1 and File S2 (refer to “README_data.txt” and “README_mcmc.txt”, respectively, for details).

## Results

### Simulation study

**Figure 1:**
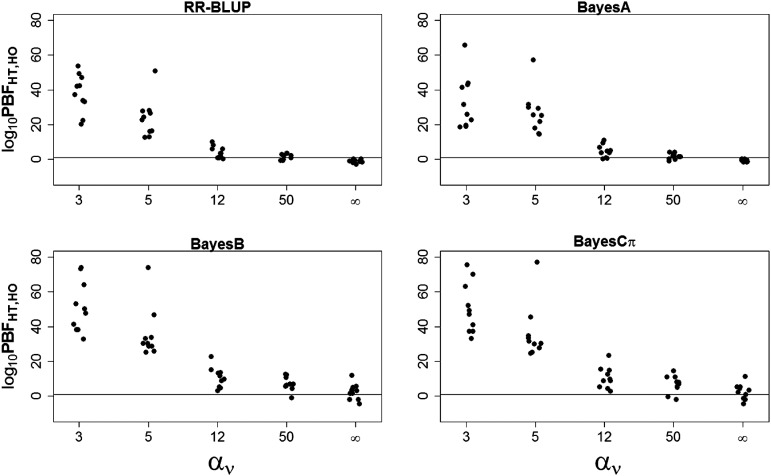
Assessment of global fit, expressed as log10 pseudo-Bayes factor (PBF), between heteroskedastic and homoskedastic whole-genome prediction (WGP) models, namely RR-BLUP, BayesA, BayesB, and BayesCπ, for 10 replicated data sets from each of five simulated scenarios defined by either residual homoskedasticity ($\alpha_{v}$→ ∞) or a gradient of residual heteroskedasticity ranging from high ($\alpha_{v}$= 3, 5) to moderate ($\alpha_{v}$= 12) to low ($\alpha_{v}$= 50). A horizontal reference line is provided at zero.

For each of the WGP models considered in this study, Figure 1 shows comparisons of global fit, expressed as ${\text{log}}_{10}{\text{ PBF}}_{\text{HT,HO}}$, between homoskedastic and heteroskedastic model specifications for scenarios reflecting a gradient of increasing residual heteroskedasticity. Recall that positive values of the ${\text{log}}_{10}{\text{ PBF}}_{\text{HT,HO}}$ indicate support for the heteroskedastic, as opposed to the homoskedastic error version of the corresponding WGP model. When residual heteroskedasticity was high ($\alpha_{\nu }$= 3 or 5), ${\text{log}}_{10}{\text{ PBF}}_{\text{HT,HO}}$was estimated to be between 12.8 and 77.3 across MC replicates fitted with any of the WGP models. This supports a strong advantage in global fit for heteroskedastic, rather than homoskedastic, error specifications, regardless of the specific WGP model. As the amount of residual heteroskedasticity decreased ($\alpha_{\nu }$= 12), so did the values of ${\text{log}}_{10}{\text{ PBF}}_{\text{HT,HO}}$ and thus the relative advantage of the heteroskedastic error WGP model over its homoskedastic error counterpart. Under scenarios of low heteroskedasticity ($\alpha_{\nu }$= 50), or of homogeneous residual variance ($\alpha_{\nu }$→ ∞), the values of ${\text{log}}_{10}{\text{ PBF}}_{\text{HT,HO}}$ under RR-BLUP and BayesA were closer to zero, thus indicating no apparent advantage of heteroskedastic WGP models over their homoskedastic error counterparts; in turn, BayesB and BayesCπ showed greater uncertainty in these conditions. Overall, we note that, when the amount of residual heteroskedasticity in the data was high ($\alpha_{\nu }$= 3 or 5), ${\text{log}}_{10}{\text{ PBF}}_{\text{HT,HO}}$ consistently selected the appropriate heteroskedastic error specification for all WGP models; however, as mentioned above, the discriminatory capability of ${\text{log}}_{10}{\text{ PBF}}_{\text{HT,HO}}$ to detect random sources of residual heteroskedasticity was partially, though incrementally, impaired when heteroskedasticity was moderate ($\alpha_{\nu }$= 12) or low ($\alpha_{\nu }$= 50).

Table 1 presents a summary of the posterior inference on the hyperparameter $\alpha_{\nu }$defining the degree of heterogeneity of residual variance across levels of the random effect factor for 10 MC replicates under each of the simulation scenarios fitted with the heteroskedastic WGP models. Coverage probabilities for $\alpha_{\nu }$, defined as proportion MC replicates for which the 95% highest posterior density (HPD) included the true parameter value, under WGP models RR-BLUP and BayesA were mutually identical at 100%, 90%, 100%,100% when true $\alpha_{\nu }$= 3, 5, 12, 50, respectively. In turn, coverage probabilities were 100%, 90%, 70%, 20% for BayesB and 100%, 90%, 80%, 20% for BayesCπ, when true $\alpha_{\nu }$= 3, 5, 12, 50, respectively. As might be expected, inferential precision on $\alpha_{\nu }$was maximized when heteroskedasticity was high, as indicated by the smallest difference between minimum and maximum values of the lower and upper boundaries of the 95% HPD on $\alpha_{\nu }$ (Table 1). The reverse was also true, as inference on $\alpha_{\nu }$was most uncertain when heteroskedasticity was not present. That is, enhanced model fit of a heteroskedastic WGP model relative to its homoskedastic counterpart under conditions of heterogeneous variances may be explained by increased precision of inference on the hyperparameter$\alpha_{\nu }$, and vice versa. These results on posterior inference of the heteroskedasticity parameter $\alpha_{\nu }$ are consistent with those presented for overall goodness of fit in Figure 1.

**Table 1:** Posterior inference on the hyperparameter $\alpha_{v}$ for simulated datasets

True $\alpha_{v}$	RR-BLUP		BayesA		BayesB		BayesCπ	
	${\tilde{\bar{\alpha }}}_{v}$	(minL, maxU)	${\tilde{\bar{\alpha }}}_{v}$	(minL, maxU)	${\tilde{\bar{\alpha }}}_{v}$	(minL, maxU)	${\tilde{\bar{\alpha }}}_{v}$	(minL, maxU)
3	3.83	(1.55, 7.94)	3.63	(1.66, 8.57)	3.37	(1.50, 7.65)	3.43	(1.47, 7.53)
5	6.94	(1.45, 19.84)	6.59	(1.48, 23.89)	4.54	(1.28, 11.74)	4.58	(1.31, 11.87)
12	22.74	(3.31, 301.75)	28.07	(3.48, 307.89)	10.70	(2.82, 27.77)	11.03	(2.83, 28.23)
50	116.11	(3.78, 586.82)	99.05	(4.04, 526.37)	11.96	(3.64, 106.07)	12.26	(3.62, 174.62)
∞	224.67	(3.71, 1363.73)	315.31	(3.85, 1324.12)	16.16	(3.56, 149.61)	16.67	(3.47, 136.10)

Median of the posterior mean of $\alpha_{v}$(${\tilde{\bar{\alpha }}}_{v}$) [as well as minimum and maximum values for the respective lower and upper boundaries (minL, maxU) of the 95% highest posterior density intervals of the posterior distribution of $\alpha_{v}$] based on 10 Monte Carlo replicates across simulation scenarios consisting of a gradient of increasing heteroskedasticity. Simulated data were fitted with heteroskedastic specifications of whole-genome prediction models, namely RR-BLUP, BayesA, BayesB, and BayesCπ.

To validate inferential performance on the fixed effect parameters **τ** specified on the residual variance, we considered the posterior density of the ratio of $\tau_{1}$ over $\tau_{2}$. Coverage probability of the 95% HPD for the true value of the parameter ratio under heteroskedastic WGP models was 92% for both BayesB and BayesCπ, and 94% for both RR-BLUP and BayesA across simulation scenarios. In all cases, the observed coverage was within probabilistic expectation.

**Figure 2:**
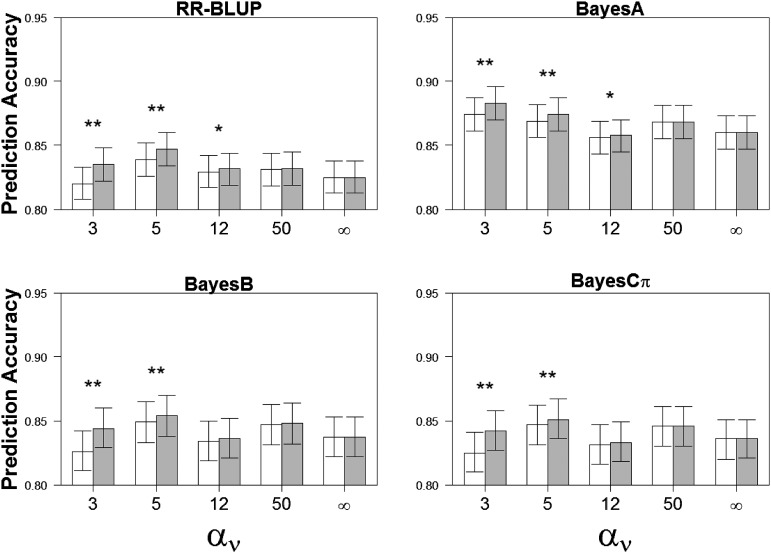
Genomic prediction accuracy (least square mean estimates and 95% confidence intervals) for heteroskedastic (gray) and homoskedastic (white) specifications of WGP models considered in this study, namely RR-BLUP, BayesA, BayesB, and BayesCπ, under simulation scenarios defined by either residual homoskedasticity ($\alpha_{v}$→ ∞) or a gradient of residual heteroskedasticity ranging from high ($\alpha_{v}$= 3, 5) to moderate ($\alpha_{v}$= 12) to low ($\alpha_{v}$= 50). Genomic prediction accuracy was defined as the Pearson correlation coefficient between true breeding value and expected breeding value. ** and * indicate differences between heteroskedastic and homoskedastic versions of each WGP model at *P* = 0.001 and *P* = 0.05, respectively.

Estimated genomic prediction accuracies (and corresponding standard errors) of heteroskedastic and homoskedastic error versions of WGP models are shown in Figure 2. For all RR-BLUP, BayesA, BayesB and BayesCπ models, the heteroskedastic specification showed a gain on genomic prediction accuracy relative to the homoskedastic WGP counterpart whenever the amount of residual heteroskedasticity in the data were high (*i.e.*, $\alpha_{\nu }$= 3 or 5, *P* < 0.001 in all cases). However, no evidence for any predictive advantage of heteroskedastic WGP specifications was apparent if the data had been generated under conditions of low or null heteroskedasticity (*i.e.*, $\alpha_{\nu }$= 50 or ∞; *P* > 0.30 in all cases). For situations of moderate heteroskedasticity (*i.e.*, $\alpha_{\nu }$= 12) fitted with RR-BLUP or BayesA WGP models, the heteroskedastic error specification yielded greater (*P* < 0.05) genomic predictive accuracy than its homoskedastic counterpart but this difference was not apparent using variable selection models like BayesB or BayesCπ. Despite the significant increase in genomic prediction accuracy by heteroskedastic WGP models when the data were highly heteroskedastic, we note that the gain in accuracy relative to the homoskedastic specification was of a relatively small magnitude (*i.e.*, range from 0.009 to 0.018 for $\alpha_{\nu }$= 3 and from 0.005 to 0.008 for $\alpha_{\nu }$= 5 across MC replicated data sets).

**Figure 3:**
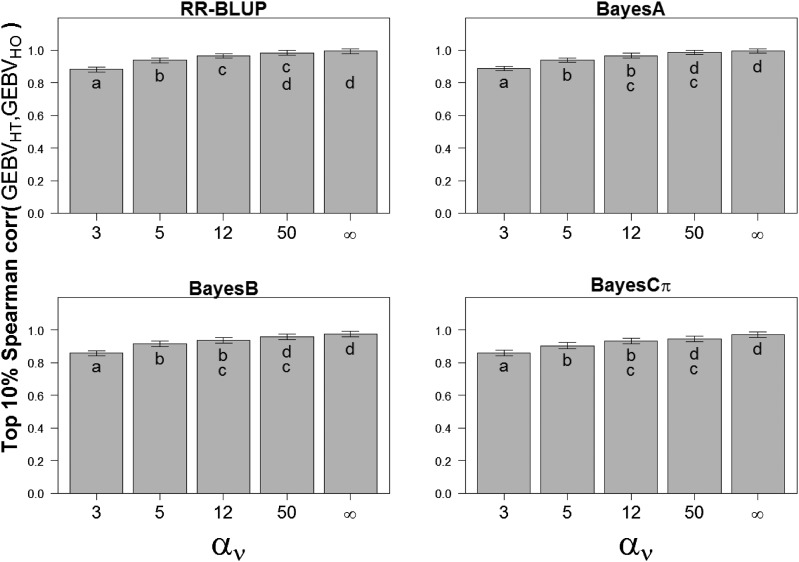
Spearman’s correlation coefficient (least square mean estimates and 95% confidence intervals) between top 10% homoskedastic-assuming predicted genomic breeding values (GEBV), and their heteroskedastic-assuming counterpart GEBV across WGP models under simulation scenarios defined by either residual homoskedasticity ($\alpha_{v}$→ ∞) or a gradient of residual heteroskedasticity ranging from high ($\alpha_{v}$= 3, 5) to moderate ($\alpha_{v}$= 12) to low ($\alpha_{v}$= 50). (a,b,c,d) Different letters indicate significant differences in ranking at α = 0.05.

To further characterize potential practical implications of heteroskedastic *vs.* homoskedastic WGP models in the context of breeding programs, we explored differences in the ranking of individuals of extreme genetic merit. We computed the Spearman correlation of the top 10% individuals whose GEBV had been estimated from homoskedastic and heteroskedastic WGP models across the simulated gradient of residual heteroskedasticity (Figure 3). Results on the bottom 10% ranked individuals showed a similar pattern and are thus not discussed further. For homoskedastic scenarios ($\alpha_{\nu }$→ ∞) or scenarios of low heteroskedasticity ($\alpha_{\nu }$= 50), the Spearman rank correlation between homoskedastic- and heteroskedastic-based GEBVs from RR-BLUP, BayesA, BayesB or BayesCπ WGP models for the top 10% animals was close to 1, thus indicating minor concerns for selection purposes. However, as the amount of heteroskedasticity increased, the Spearman correlation between heteroskedastic-based GEBV and homoskedastic-assuming GEBV for the top 10% individuals decreased to an estimated value of 0.85. This result suggests nonnegligible reranking of top individuals for selection purposes. Given response to selection, this finding could potentially have direct implications for breeding programs despite the small magnitude of the overall gain on genomic prediction accuracy described before.

### MSU swine resource population data

For carcass temperature at 45 min postmortem, the variance ${\text{σ}}_{u}^{2}$ of polygenic effects were estimated at 0.022 and 0.036 for homoskedastic and heteroskedastic error specifications, respectively. For loin muscle pH at 45 min, the corresponding estimates of ${\text{σ}}_{u}^{2}$ were 0.006 and 0.005, respectively.

We first assessed evidence for residual heteroskedasticity on the traits carcass temperature and loin muscle pH 45 min postmortem selected for this study from the MSU resource population. Table 2 summarizes the posterior inference for $\alpha_{\nu }$ in the heteroskedastic specification of WGP models on the complete dataset. Recall that the hyperparameter $\alpha_{\nu }$ defines the magnitude of non-systematic heterogeneity of residual variances for each of the selected traits as a function of slaughter dates clusters. For carcass temperature at 45 min postmortem, the posterior mean of $\alpha_{\nu }$was smaller than two in all cases. Similarly, the magnitude of the upper boundaries of the 95% HPD on $\alpha_{\nu }$ did not exceed three under any of the heteroskedastic WGP models considered, thereby providing evidence for strong cluster-based heterogeneity of residual variances for this trait. In fact, the range of posterior means of slaughter date-specific $v_{l}$ for carcass temperature at 45 min postmortem was greater than eight-fold, thereby indicating that the residual variance for some slaughter dates clusters was estimated to be as large as eight times greater than the residual variance in other slaughter clusters. In turn, for loin muscle pH at 45 min postmortem, the posterior mean of $\alpha_{\nu }$ was below six, and the corresponding upper boundaries of its 95% HPD was approximately 10 in all cases, thus indicating milder cluster-driven residual heteroskedasticity for this trait, whereby the range of posterior means of $v_{l}$was close to three-fold across all 33 slaughter dates clusters. These results are consistent with our findings during preliminary data screening.

**Table 2:** Posterior inference on the hyperparameter $\alpha_{v}$ for two quantitative traits from the Michigan State University swine resource population

	Carcass Temperature at 45 Min		Loin Muscle pH at 45 Min	
	${\bar{\alpha }}_{v}$	95% HPD	${\bar{\alpha }}_{v}$	95% HPD
RR-BLUP	1.66	(1.05, 2.45)	5.14	(2.09, 8.90)
BayesA	1.62	(1.06, 2.43)	5.01	(2.03, 8.59)
BayesB	1.89	(1.09, 2.86)	5.76	(2.27, 9.94)
BayesCπ	1.88	(1.11, 2.84)	5.84	(2.26, 10.01)

Posterior mean for $\alpha_{v}$ (${\bar{\alpha }}_{v}$), as well as 95% highest posterior density (HPD) intervals, based on heteroskedastic whole-genome prediction models, namely RR-BLUP, BayesA, BayesB, and BayesCπ, are presented.

We also explored the effect of sex as an additional source of heteroskedasticity on the selected traits, as represented by parameter **τ** in Equation (2). Based on the set-to-zero parameterization implemented in this study, the parameter **τ** may be interpreted as a ratio of female-to-male residual variances, whereby a ratio of one indicates homogeneous residual variances for both sexes. For carcass temperature at 45 min postmortem, the 95% HPD of **τ** fitted with any of the WGP models ranged from a lower boundary of 0.7 to an upper boundary of 1.2. For loin muscle pH, the corresponding 95% HPD range was 0.9 to 1.5. It then follows no evidence for sex-based heteroskedasticity of either trait regardless of WGP model.

Next, we consider relative global fit of homoskedastic *vs.* heteroskedastic error WGP models to the actual data using PBF (Gelfand 1996), and use a threshold value of ${\text{log}}_{10}{\text{ PBF}}_{\text{HT,HO}}=2$ to conclude upon a decisive difference in fit between models (Kass and Raftery 1995). For carcass temperature at 45 min postmortem, the range of ${\text{log}}_{10}{\text{ PBF}}_{\text{HT,HO}}$ across the five cross-validation folds was [45.7, 81.3] for RR-BLUP, [46.9, 81.0] for BayesA, [44.3, 77.5] for BayesB, and [43.8, 76.9] for BayesCπ WGP models. In turn, for loin muscle pH at 45 min postmortem, the range of ${\text{log}}_{10}{\text{ PBF}}_{\text{HT,HO}}$was [9.7, 14.4], [9.8, 14.6], [8.8, 13.7] and [8.6, 13.8]. These results favor the use of heteroskedastic WGP error models for both traits, and under all WGP specifications considered here. The larger magnitude of ${\text{log}}_{10}{\text{ PBF}}_{\text{HT,HO}}$, and hence greater evidence of residual heteroskedasticity for carcass temperature relative to loin muscle pH, is consistent both with our preliminary screening and with our posterior inference on $\alpha_{\nu }$ as described previously.

We also assessed predictive performance of homoskedastic and heteroskedastic WGP models. We first conducted a sensitivity analysis to evaluate the stability of cross-validation predictive ability using BayesCπ under different choices of $s_{g}^{2}$ as $4.95\times {\text{10}}^{-5}$, $4.95\times 10^{-7}$and $4.95\times {\text{10}}^{-10}$for carcass temperature and $2.01\times {\text{10}}^{-6}$, $2.01\times {\text{10}}^{-8}$and $2.01\times {\text{10}}^{-11}$for loin muscle pH trait. Similarly, sensitivity assessments were also conducted for BayesB looking at choices of $s_{g}^{\text{2}}$ as $6.35\times {\text{10}}^{-5}$, $6.35\times {\text{10}}^{-7}$ and $6.35\times {\text{10}}^{-10}$for carcass temperature and $2.64\times {\text{10}}^{-6}$, $2.64\times {\text{10}}^{-8}$ and $2.64\times {\text{10}}^{-11}$ for loin muscle pH trait. As expected, changes in the specification of $s_{g}^{\text{2}}$ were compensated with changes in the estimates of the proportion of SNP markers with nonzero effects (Yang *et al.* 2015). In turn, the estimated median cross-validation prediction accuracies for carcass temperature, and its corresponding standard deviation, across cross-validation folds at any choice of $s_{g}^{\text{2}}$ were 0.86 ± 0.05 for homoskedastic error BayesCπ or BayesB and 0.86 ± 0.04 for heteroskedastic error BayesCπ or BayesB. For loin muscle pH, cross-validation prediction accuracy based on homoskedastic error BayesCπ or BayesB ranged from 0.31 ± 0.06 to 0.29 ± 0.07 across prior specifications of $s_{g}^{\text{2}}$. For heteroskedastic error BayesCπ or BayesB, cross-validation predictive ability ranged from 0.32 ± 0.07 to 0.29 ± 0.07 across values of $s_{g}^{\text{2}}$. Overall, sensitivity analyses assessment indicated little reason to be concerned about specification of hyperparameters for the purpose of prediction accuracy.

**Figure 4:**
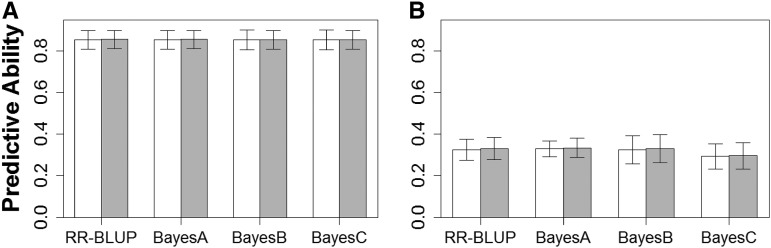
Estimated cross-validation predictive ability (and 95% confidence intervals) of genomic breeding values for (A) carcass temperature 45 min postmortem and (B) loin muscle pH 45 min postmortem fitted with heteroskedastic (gray bars) and homoskedastic (white bars) specifications of WGP models considered in this study, namely RR-BLUP, BayesA, BayesB, and BayesCπ. Cross-validation predictive ability is represented by the Pearson correlation coefficient between observed phenotypes in the validation fold, and their corresponding predictions based on estimates from the training folds in a five-fold cross-validation study.

Figure 4 depicts estimated cross-validation predictive abilities across five folds for both carcass temperature and loin muscle pH at 45 min postmortem. Across WGP models, cross-validation predictive abilities for carcass temperature and loin muscle pH 45 min postmortem were estimated to be approximately 0.85 and 0.32, respectively. For neither trait did we find any evidence for differences in cross-validation predictive ability between homoskedastic *vs.* heteroskedastic specifications of any of the WGP models considered (*P* > 0.25 in all cases for either trait).

Often in animal breeding, greater interest is directed toward animals that exhibit extreme GEBV, as they are the ones likely to be selected as parents for the next generation. Table 3 reports Spearman’s rank correlation coefficient between homoskedastic-error *vs.* heteroskedastic-error GEBV for animals of extreme genetic merit. For both traits, and regardless of WGP model, we observed considerable reranking of the top and bottom 10% individuals, particularly as the degree of residual variance heterogeneity in the data increased. In fact, for loin muscle pH at 45 min postmortem, the corresponding estimated median rank correlations ranged from 0.52 to 0.70 (top), and from 0.64 to 0.70 (bottom) across WGP models; in turn, for the more heteroskedastic trait (*i.e.*, carcass temperature at 45 min postmortem), the median rank correlation of GEBV for top and bottom 10% animals ranged from 0.05 to 0.38 (top) and from 0.43 to 0.54 (bottom) across WGP models. Such variability in rankings of GEBV may be partially due to the relatively small sample size (*i.e.*, only 10% animals within a cross-validation fold) used to estimate the rank correlation coefficient. This is further supported by the considerable variability observed among cross-validation folds in the reranking of individuals based on homoskedastic-based GEBV relative to their heteroskedastic counterpart, though this variability was particularly noticeable for carcass temperature. Again, this may be partially explained by the relatively larger magnitude of residual heteroskedasticity detected for this trait. We further note that reranking of extreme GEBV using homoskedastic *vs.* heteroskedastic errors seemed to be particularly extreme when the BayesB WGP specification was implemented.

**Table 3:** Estimated Spearman’s rank correlation coefficient between homoskedastic-based and heteroskedastic-based estimated genomic breeding values corresponding to the top and bottom 10% individuals (approximately 17–20 within a cross-validation fold) for two quantitative traits of swine data

Traits		RR-BLUP		BayesA		BayesB		BayesCπ	
		$\tilde{\rho }$	(min, max)	$\tilde{\rho }$	(min, max)	$\tilde{\rho }$	(min, max)	$\tilde{\rho }$	(min, max)
Carcass temperature 45 min	Top	0.17	(0.03, 0.65)	0.28	(0.12, 0.63)	0.05	(–0.16, 0.69)	0.38	(0.06, 0.46)
	Bottom	0.54	(0.15, 0.62)	0.44	(0.19, 0.59)	0.43	(0.27, 0.74)	0.52	(0.39, 0.89)
Loin muscle pH 45 min	Top	0.70	(0.46, 0.79)	0.64	(0.33, 0.82)	0.52	(0.18, 0.74)	0.66	(0.46, 0.81)
	Bottom	0.70	(0.57, 0.79)	0.69	(0.44, 0.81)	0.64	(0.22, 0.77)	0.70	(0.60, 0.83)

Median rank correlation ($\tilde{\rho }$) between predicted genomic breeding values, as well as minimum and maximum estimates, across five cross-validation folds.

**Figure 5:**
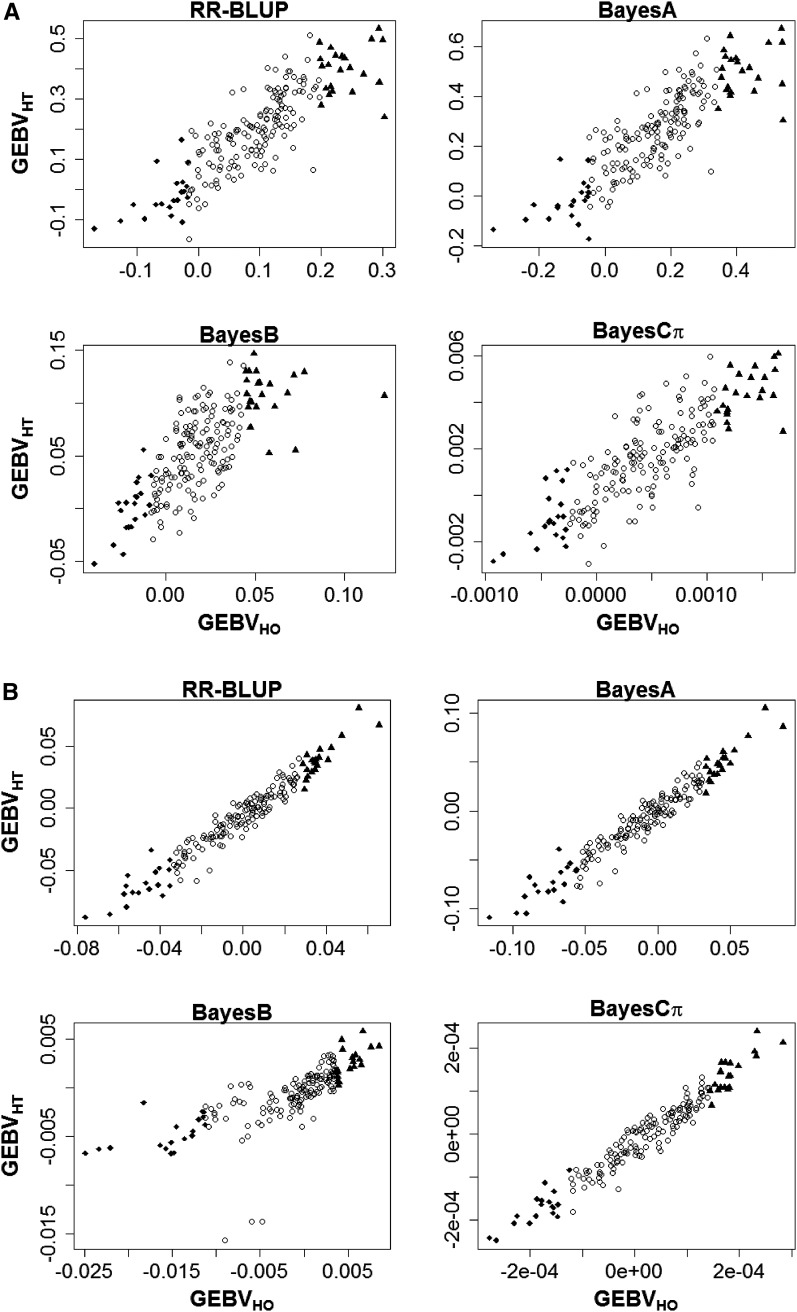
Illustrative scatter plots of predicted genomic breeding values obtained from homoskedastic (HO) and heteroskedastic (HT) specifications of RR-BLUP, BayesA, BayesB, and BayesCπ WGP models fitted to carcass temperature (A) and loin muscle pH (B) at 45 min postmortem. Symbols indicate individuals ranked among the top 10% (▴), bottom 10% (♦) or remainder (○) based on the homoskedastic WGP model. Plots are based on a single cross-validation fold and are meant for illustration only.

For further illustration, we selected a cross-validation fold and depicted a scatterplot of homoskedastic-error *vs.* heteroskedastic-error GEBV for carcass temperature (Figure 5A) and for loin muscle pH (Figure 5B) under each WGP model. For both traits, individuals that showed extreme genetic merit under homoskedastic error assumptions had their GEBV considerably attenuated when residual heteroskedasticity was accounted for (*e.g.*, Figure 5A, BayesB). In fact, an individual with extremely high GEBV inferred under a homoskedastic error model may not be considered as a viable selection candidate if its GEBV was estimated from a heteroskedastic error WGP model. This was indeed the case for the two individuals with top homoskedastic-based GEBV in the complete dataset. It is interesting to note that these top two individuals derived from one slaughter date cluster that had the largest posterior mean for the relative residual variance $v_{l}$ (data not shown). Conversely, candidate individuals with top or bottom genetic merit may be overlooked by using conventional homoskedastic error models (*e.g.*, Figure 5B, BayesB).

Taken together, the observed reranking could have practical implications from a selection point of view. In support of this observation, we note that the mean of genomic breeding values in the top 10% individuals was between 1.2× (based on BayesA) to 10× (based on BayesCπ) greater in magnitude when estimated based on the heteroskedastic WGP model relative to the homoskedastic specification for either trait. Similar results were observed for the bottom 10% individuals with extreme genetic merit based on the hetero- *vs.* homoskedastic WGP specification. It should be acknowledged, though, that these comparisons are conditioned on the models used to predict mean genomic breeding values.

## Discussion

In this study, we extend classical WGP models to account for potential heterogeneous residual variances across environments, and further assess whether explicit accounting for such heteroskedasticity may impact accuracy of prediction of genomic breeding values.

Environmental residual heteroskedasticity is a rather common phenomenon across agricultural environments in livestock production. For instance, residual variance estimates for birth weight in an Italian Piemontese cattle population differed by approximately 10-fold across herds (Kizilkaya and Tempelman 2005), and that of average daily gains in feedlot cattle from the US Midwest differed by more than 12-fold across contemporary groups (Cernicchiaro *et al.* 2013). Backfat thickness in pigs was shown to display considerable residual heteroskedasticity based on an animal model, whereby residual variances ranged by approximately eight-fold across herds (See 1998). Similarly, in this study, we found evidence for considerable environmentally-driven heterogeneity of residual variances in other swine carcass traits, as indicated by the small magnitude of posterior means of $\alpha_{\nu }$ for carcass temperature and loin muscle pH. These results indicate considerable departure from the residual homoskedasticity assumption commonly invoked by standard WGP models.

Gianola and Rosa (2015) adverted that modeling heterogeneous residual variances across environments was likely to be important for reliable genomic selection, as further supported by our results. Unaccounted-for heteroskedastic errors can potentially impact breeding decisions as animals from the most diverse environments might then be disproportionally selected (Hill 1984). Indeed, previous studies have shown nonnegligible reranking of top and bottom 10% progenitors when heterogeneity of residual variances across environment or management groups is properly modeled (Cardoso *et al.* 2005; Kizilkaya and Tempelman 2005). In fact, incorrectly assuming homogeneous residual variances could cause a substantial reduction in selection efficiency, particularly under conditions of low heritability (Garrick and VanVleck 1987). Heritabilities of most technological quality traits of meat in swine, such as the ones evaluated in this study, has been reported to range from low to moderate, as the average value for many studies fall into the range 0.10–0.30 (reviewed by Sellier and Monin 1994 and Ciobanu *et al.* 2011).

Based on this evidence, we extended homoskedastic WGP models to allow for environmental heterogeneity of residual variances, and evaluated the relative performance of heteroskedastic and homoskedastic error WGP models in terms of both global fit and predictive performance. Our simulation study showed considerable improvements in global model fit when extreme residual heteroskedasticity was properly accounted for, though the advantage of heteroskedastic error WGP models seemed to dissipate quickly for even moderate amounts of environmental heterogeneity in residual variances, particularly under WGP models without variable selection (*i.e.*, RR-BLUP and BayesA). Furthermore, the observed advantage of heteroskedastic error WGP models in global fit translated into very small (∼1–2%), albeit significant, gains in genomic prediction accuracy under conditions of extreme data heteroskedasticity. As the amount of residual heteroskedasticity decreased, so did the power to detect differences in genomic prediction accuracy between heteroskedastic and homoskedastic model specification. This was particularly noticeable for WGP models with variable selection (*i.e.*, BayesB or BayesCπ) relative to those without variable selection (*i.e.*, RR-BLUP and BayesA). For the specific data application used in this study corresponding to the MSU swine resource population, there was no evidence of any gains in cross-validation predictive ability for selected carcass traits when heterogeneous residual variances across environments were explicitly modeled. This finding was somewhat unexpected given the extreme level of environmental heterogeneity of residual variances observed in at least one of those traits. The high level of environmental heteroskedasticity in the carcass temperature trait was recognized both by posterior inference on the hyperparameter $\alpha_{v}$ and by improved global model fit of the heteroskedastic error model relative to the homoskedastic error model. Yet, it is possible that additional gains in prediction accuracy from specifying heteroskedasticity, either of environmental or genetic origin, may be difficult to observe due to the already large magnitude of “baseline” cross-validation predictive ability for this trait (∼0.85) based on standard homoskedastic error WGP models.

It is unclear whether a genetic component might have contributed to the high level of residual heteroskedasticity observed in the carcass temperature trait. A recent study by Yang *et al.* (2011) explored the use of parametric genomic models that specify genetic control of environmental variance in a swine production system. In particular, classical WGP models were extended to assess putative marker effects not only on GEBV but also on environmental variability. Consistent with our results, that study indicated enhanced fit of heteroskedastic error models. However, the gains in accuracy of prediction were either of small magnitude in simulated data or not at all apparent when applied to back fat thickness data in pigs, as was also observed in our application. Additional statistical methods for detecting genetic loci affecting phenotypic variability were recently introduced; proposed approaches range from fully-parametric (Rönnegård and Valdar 2011; Yang *et al.* 2011) through classical nonparametric (Paré *et al.* 2010; Struchalin *et al.* 2010), including a two-stage semi-parametric approximation (Hill and Mulder 2010). Following a thorough review, Rönnegård and Valdar (2012) highlighted the inferential importance of simultaneous estimation of effects on mean and variance as a strength of parametric methods for modeling variance-controlling QTL.

Ideally, heterogeneous genetic and residual variances should be modeled simultaneously, for example with multiple breed studies (de Roos *et al.* 2009) or genotype by environment interaction (Edwards and Jannink 2006; Jarquin *et al.* 2014; Lopez Cruz *et al.* 2015) studies, both of which require the specification of heterogeneous genetic variances. A study by Chen *et al.* (2014) explored multi-population genomic prediction for milk production traits on two dairy breeds using a multi-task Bayesian learning model. When error variances and marker effects variances were explicitly specified as heterogeneous across breeds (as opposed to assumed homogeneous), gains in prediction accuracy across traits ranged from 0.04 to 0.14 using a 50K SNP panel, and from 0.02 to 0.11. Also, a yield variety trial of 40 oat genotypes across 34 environments reported “stabilized” genetic predictions (*i.e.*, higher repeatability) when genetic and environmental sources of heterogeneity were explicitly specified on residual and genotype-by-environment variance components (Edwards and Jannink 2006).

It is often the case that deregressed expected breeding values are weighted and used as response variables in WGP models (Garrick *et al.* 2009) instead of actual phenotypes. In a way, modeling of weighted deregressed expected breeding values may be considered an approach to account of heterogeneous variance, in this case the variance of the expected breeding value. However, this approach may be considered *ad hoc* as its effectiveness depends on several factors such as the number of repeated measured observations, size of training data and the reliability of the breeding values (Garrick *et al.* 2009; Ostersen *et al.* 2011; Boddhireddy *et al.* 2014).

Despite the observed lack of any appreciable gain in overall accuracy of prediction of carcass trait phenotypes by heteroskedastic WGP models, the differential ranking of animals with the 10% most extreme genetic merit suggest important practical implications for the assumption of homogeneous residual variance. Substantial reranking was apparent for these candidate animals depending on whether environmental residual heteroskedasticity was explicitly accounted for in obtaining their breeding values. In fact, noticeable differences in the mean of genomic breeding values for individuals of extreme genetic merit were apparent based on heteroskedastic *vs.* homoskedastic WGP specifications. To reconcile these results, we notice that the magnitude of the difference in GEBV between most extreme individuals and the rest seems to be rather small, and thus difficult to detect, particularly given the relatively narrow range of GEBV observed for the selected traits in this population. In turn, a localized performance metric, such as the Spearman correlation coefficient among the top and bottom 10% individuals, could detect regional patterns that may not be necessarily apparent from overall performance metrics, such as cross-validation predictive ability. However, we acknowledge that, given the low-to-moderate heritabilities of the traits evaluated in this study, it is not possible to discard nonnegligible sampling variability on the estimates of Spearman correlation coefficients or other statistical reasons related to unstable behavior of correlations within extreme tails.

The very low magnitude of the posterior mean of $\alpha_{\nu }$observed in the swine data application indicates that residual variability is not homogeneous across environmental subclasses. However, extreme within-cluster residuals, for example, due to preferential treatment, may be a reasonable concern even after accounting for residual heteroskedasticity. Biased prediction of breeding values is a problem often encountered under conditions of preferential treatment (Kuhn and Freeman 1995). Our observation of substantial reranking of extreme breeding values suggests that heteroskedastic WGP models may, at least partially, offset prediction bias due to preferential treatment. Yet, preferential treatment is a concern that further motivates the need to extend heteroskedastic error WGP models to allow for outlier robustness, as advocated by Gianola and Rosa (2015). In a nongenomic application, Cardoso *et al.* (2007) extended the univariate *t*-models proposed by Stranden and Gianola (1999) to attenuate adverse effects of preferential treatment and specified Student-*t* distributed residual heteroskedasticity across environments to potentially accommodate a more robust analysis capable of muting the influence of extreme observations on inferences of cluster specific residual variances. Given the breeding objective of ranking candidates for selection, a heavy-tailed residual distribution combined with explicit modeling of environmental heteroskedasticity is likely to yield more robust genomic predictions in the sense of reducing influence from outlying identifiable clusters and extreme individual datapoints.

Most recently, WGP models have been used to predict complex human traits, such as risk of disease and life expectancy (de los Campos *et al.* 2012; Vazquez *et al.* 2012). One can surmise that environmentally-driven heteroskedasticity is likely present in this context as well, though the full extent of it remains unclear. Predictive performance of WGP models for human traits can be low, mostly due to factors unique to human populations, such as unrelatedness of individuals and short LD patterns (de los Campos *et al.* 2013b). Extending WGP models for complex human traits to explicitly model heterogeneous residual variances across environments could potentially help account for still-unexplained variance, and thus affect the extent of missing heritability in human populations.

Finally, WGP models often require estimation of a large number of SNP marker effects and considerations for computing efficiency become paramount, particularly since MCMC inference can be computationally expensive. Computational enhancements for homoskedastic WGP models have been developed based on expectation-maximization (EM) based algorithms (Hayashi and Iwata 2010), or analytically derived posterior densities of each marker effect (Meuwissen *et al.* 2009). Heteroskedastic error extensions to WGP models, such as those presented here, could also be further modified to enhance computational efficiency. For example, an EM-like algorithm such as that proposed by Gianola *et al.* (1992) may be adapted to obtain empirical Bayes estimates of environment-specific variances in a WGP context.

### Conclusions

In this study, we describe extensions to classical whole-genome prediction models that incorporate modeling of heterogeneous residual variances across environments and evaluate potential impact of specifying heteroskedastic *vs.* homoskedastic error models on the accuracy of prediction of genomic breeding values. Heteroskedastic error models were overwhelmingly supported by improved global fit to the data. The advantages of heteroskedastic error WGP models on overall predictive ability of carcass traits in pigs was of small magnitude, if at all present; however, considerable reranking of individuals with extreme genetic merit was observed when heteroskedasticity was explicitly accounted for. Heteroskedastic error WGP modeling should be carefully considered in breeding programs as environmental residual heteroskedasticity seems prevalent and, if unaccounted, can have considerable practical implications for selection of individuals of extreme genetic merit. Additional work tackling simultaneous modeling of heterogeneous genetic variances jointly with heterogeneous error variances and potential outlier robustness extensions is warranted.

## 
